# Profiling of the metabolic transcriptome via single molecule molecular inversion probes

**DOI:** 10.1038/s41598-017-11035-0

**Published:** 2017-09-12

**Authors:** Tessa de Bitter, Carlijn van de Water, Corina van den Heuvel, Carolien Zeelen, Astrid Eijkelenboom, Bastiaan Tops, Egbert Oosterwijk, Dimitar Kolev, Peter Mulders, Mark ter Laan, Sanne van Lith, William Leenders

**Affiliations:** 1 Dept of Pathology, Radboud University Medical Centre, Nijmegen, The Netherlands; 2 Dept of Urology, Radboud University Medical Centre, Nijmegen, The Netherlands; 3 Dept of Neurosurgery, Radboud University Medical Centre, Nijmegen, The Netherlands

## Abstract

Cancer-specific metabolic alterations are of high interest as therapeutic targets. These alterations vary between tumor types, and to employ metabolic targeting to its fullest potential there is a need for robust methods that identify candidate targetable metabolic pathways in individual cancers. Currently, such methods include ^13^C-tracing studies and mass spectrometry/ magnetic resonance spectroscopic imaging. Due to high cost and complexity, such studies are restricted to a research setting. We here present the validation of a novel technique of metabolic profiling, based on multiplex targeted next generation sequencing of RNA with single molecule molecular inversion probes (smMIPs), designed to measure activity of and mutations in genes that encode metabolic enzymes. We here profiled an isogenic pair of cell lines, differing in expression of the Von Hippel Lindau protein, an important regulator of hypoxia-inducible genes. We show that smMIP-profiling provides relevant information on active metabolic pathways. Because smMIP-based targeted RNAseq is cost-effective and can be applied in a medium high-throughput setting (200 samples can be profiled simultaneously in one next generation sequencing run) it is a highly interesting approach for profiling of the activity of genes of interest, including those regulating metabolism, in a routine patient care setting.

## Introduction

In the past four decades an overwhelming amount of data has become available on the molecular events that underlie carcinogenesis. Research has mainly focused on molecular alterations and their consequences for, among others, the PI3K/pAKT/mTOR pathway^1–4^ and cell cycle control, apoptosis^5, 6^ and DNA repair pathways^7, 8^. Currently, numerous FDA-approved drugs are available that target cancer cells based on these genetic defects with a level of specificity that is not attainable with conventional chemotherapies^9, 10^, permitting personalized medicine. Whereas targeted cancer therapies may prolong survival, it is now widely recognized that inherent genetic instability and tumor plasticity ultimately leads to therapy resistance of most cancers^11–14^.

Whatever the underlying oncogenic mutations, for proliferation cancer cells need to generate ATP to maintain energy balance and ion homeostasis, import carbon and nitrogen sources for synthesis of amino acids, nucleotides and lipids^15, 16^ and maintain redox potential to protect cells against oxidative stress^17^. Blocking one or more of these processes with specific inhibitors may prohibit proliferation and/or sensitize cells to toxic therapy in a synthetic lethality approach^18^. As an example, increasing oxidative stress in a cancer with metabolic inhibitors (e.g. of enzymes that produce NADPH) may enhance the efficacy of radiotherapy^19^ or chemotherapy^20^. With the increasing knowledge of deranged metabolic pathways in cancer^21–24^ (adjuvant) targeting of cancer-specific metabolic pathways may be a highly interesting addition to current treatment protocols.

The best-known example of cancer-specific metabolic adaptation is aerobic glycolysis, also known as the Warburg effect^25^. Warburg tumors produce lactate from pyruvate, rather than shuttle pyruvate into the mitochondrial tricarboxylic acid (TCA) cycle. As glycolysis is inefficient in terms of ATP production, cancer cells characteristically upregulate expression of genes encoding glucose transporters GLUT1 and/or GLUT3, hexokinase 2, monocarboxylate transporters (to secrete lactate) and carbonic anhydrase-9 and -12 (to ensure pH homeostasis)^26^. Besides glucose, glutamine and fatty acids are recognized as important fuels for cancer cells that adjust expression of metabolic enzymes accordingly^27–30^.

While metabolic adaptations are mostly seen as a consequence of carcinogenesis, it has been unequivocally established that metabolic alterations can also cause cancer, examples being mutations in genes encoding succinate dehydrogenase (*SDH*, pheochromocytoma and paraganglioma), fumarate hydratase (*FH*, papillary renal cancers) and isocitrate dehydrogenase 1 and 2 (*IDH1/2*, among others acute myeloid leukemia and gliomas)^31–37^. Clear cell renal cell carcinoma (ccRCC) is also considered a metabolic cancer with metabolic alterations resulting from inactivating mutations in or epigenetic silencing of *VHL* found in ~80% of cases^38, 39^. pVHL is a major regulator of ubiquitination and breakdown of hypoxia inducible transcription factors HIF-1α and HIF-2α^38^. Mutations in the aforementioned metabolic enzymes and in *VHL* have been shown to induce epigenetic alterations that affect expression of other metabolic enzymes in an unpredictable fashion^40–43^.

To apply inhibitors of metabolism as potential additions to the current anti-tumor armamentarium, it is of high importance to identify which metabolic pathways are active in a specific cancer in a personalized fashion. Here we applied a novel next generation-sequencing based method using single molecule molecular inversion probes (smMIPs)^44^, to detect expression levels of 104 genes involved in metabolism, and concomitantly identify variants therein. As a proof of concept, we applied smMIPs to map part of the metabolic transcriptome of a VHL-defective ccRCC cell line and a VHL-rescued isogenic derivative, as well as in four clinical samples of clear cell renal cell cancers, with matching normal kidney tissue, and patient-derived glioma xenograft models. We validated the technique by correlating results with whole transcriptome RNAseq data (as gold standard for transcriptome analysis) and protein expression. We further verified the ability of the assay to detect oncogenic mutations in cell lines and patient tumor tissues.

Our data show that targeted RNA sequencing of transcripts encoding metabolic enzymes using smMIPs predict the predominant metabolic pathways that are operational in cancer and simultaneously allows variant detection in the targeted transcripts.

## Results

### SmMIP based library preparation and sequencing

**Figure 1:**
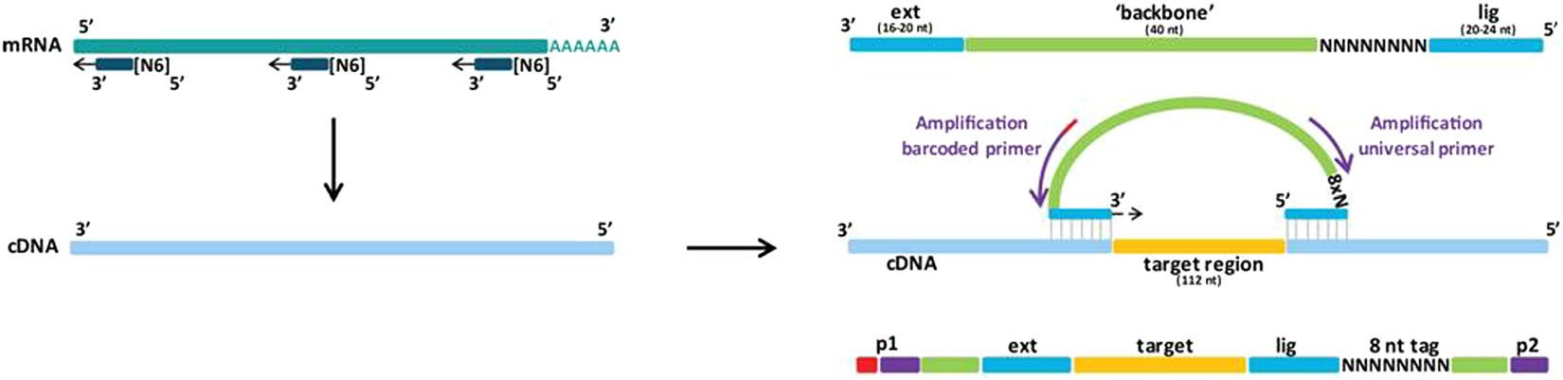
Principle of smMIP-based targeted RNA sequencing. The procedure depends on the hybridization of molecular inversion probes consisting of a ligation and an extension probe that are connected via a backbone. Capture hybridization leaves for each smMIP a gap of 112 nt that is enzymatically extended and closed by ligation. After exonuclease digestion of non-ligated probes, the remaining library of circularized smMIPS is PCR-amplified with primers in the smMIP backbone. Note that the ligation probe is flanked by a random 8N sequence that allows correction for PCR duplicates. During PCR, for each sample a unique barcode primer is used allowing identification of sample-specific reads.

SmMIP-based next generation sequencing (NGS) of genomic DNA was recently introduced in routine diagnostics in our institute to detect tumor-associated mutations in DNA^45^. To investigate whether smMIPs can also be used for multiplex determination of gene expression levels, concomitant with variant detection, we designed a smMIP set for targeted detection and sequencing of 104 transcripts encoding metabolic enzymes (see Table 1) and 18 tyrosine kinases with relevance for oncology (not shown). The technique is summarized in Fig. 1, and consists of annealing of a panel of hundreds of specifically designed smMIPs to cDNAs of interest in a sample. SmMIPs are composed of a ligation and extension probe, connected by a backbone. After hybridization of an individual smMIP to its target cDNA via its specific ligation and extension probes, a gap of 112 nt is left which is then enzymatically filled and ligated, leaving a library of circularized smMIPs. After purification and PCR with barcoded primers in the smMIP backbone, the library is subjected to next generation sequencing followed by bio-informatic analysis (see Materials and Methods for details).

**Table 1:** Metabolic transcripts for smMIP design.

Gene Symbol	Gene name	RefSeq mRNA ID (hg19)
ABAT	(4-)Aminobutyrate transaminase	NM_001127448.1
ACACA	Acetyl-CoA carboxylase alpha	NM_198834.2
ACACB	Acetyl-CoA carboxylase beta	NM_001093.3
ACLY	ATP citrate lyase	NM_001303275.1
ACO2	Acotinase 2	NM_001098.2
ACSS2	Acetyl-CoA synthetase	NM_001242393.1
ALDOA	Aldolase, fructose-bisphosphate A	NM_000034.3
ARHGAP26	Rho GTPase activating protein 26	NM_015071.4
ATG4A	Autophagy related 4 A, cysteine peptidase	NM_052936.3
ATP5A1	ATP synthase complex, F1-ATP SYNTHASE SUBUNIT	NM_001257335.1
ATP5C1	ATP synthase complex, F1-ATP SYNTHASE SUBUNIT	NM_001001973.1
BCAT1	Branched chain amino-acid transaminase 1, cytosolic	NM_001178094.1
BCAT2	Branched chain amino-acid transaminase 2, mitochondrial	NM_001284325.1
C12orf5 (TIGAR)	TP53-induced glycolysis regulatory phosphatase	NM_020375.2
CA12	Carbonic anhydrase XII	NM_206925.2
CA9	Carbonic anhydrase IX	NM_001216.2
CBR1	Carbonyl reductase 1	NM_001286789.1
CBS	Cystathione β-synthase	NM_000071.2
CHKA	Choline kinase alpha	NM_212469.1
CKB	Creatine kinase, braintype	NM_001823.4
CPT1A	Carnitine palmitoyltransferase	NM_001031847.2
CS	Citrate synthase	NM_004077.2
CYCS	Cytochrome C, somatic	NM_018947.5
D2HGDH	D-2-hydroxyglutarate dehydrogenase	NM_152783.4
EGLN1	Prolylhydroxylase	NM_022051.2
ENO1	Enolase 1, (alpha)	NM_001428.3
EPAS1	Hypoxia-inducible factor 2-alpha	NM_001430.4
FASN	Fatty acid synthase	NM_004104.4
FH	Fumarate hydratase	NM_000143.3
G6PC	Glucose-6-phosphatase, catalytic subunit	NM_000151.3
G6PD	Glucose-6-phosphate dehydrogenase	NM_000402.4
GAD1	Glutamate decarboxylase	NM_000817.2
GAPDH	Glyceraldehyde-3-phosphate dehydrogenase	NM_002046.5
GCLC	Glutamate cysteine ligase	NM_001498.3
GCLM	Glutamate cysteine ligase	NM_001308253.1
GFPT1	Fructose-6-phosphate amido-transferase	NM_001244710.1
GLDC	Glycine dehydrogenase	NM_000170.2
GLS	Glutaminase	NM_001256310.1
GLUD1	Glutamate dehydrogenase 1	NM_005271.3
GLUD2	Glutamate dehydrogenase 2	NM_012084.3
GLUL	Glutamine synthetase	NM_001033056.3
GOT1	Glutamate oxaloacetate transaminase	NM_002079.2
GPI	Glucose-6-phosphate isomerase	NM_001289789.1
GPI	Phosphoglucose isomerase = GPI	NM_001289790.1
GPT	Glutamate pyruvate transaminase	NM_005309.2
GSS	Glutathione synthetase	NM_000178.2
HIF1A	Hypoxia-inducible factor 1-alpha	NM_001530.3
HK1	Hexokinase 1	NM_000188.2
HK2	Hexokinase 2	NM_000189.4
HK3	Hexokinase 3	NM_002115.2
IDH1	Isocitrate dehydrogenase 1 (NADP + ), soluble	NM_005896.3
IDH2	Isocitrate dehydrogenase 2 (NADP + ), mitochondrial	NM_002168.3
IDH3A	Isocitrate dehydrogenase 3, mitochondrial, alpha	NM_005530.2
IDH3B	Isocitrate dehydrogenase 3, mitochondrial, beta	NM_006899.3
IDH3G	Isocitrate dehydrogenase 3, mitochondrial, gamma	NM_174869.2
L2HGDH	L-2-hydroxyglutarate dehydrogenase	NM_024884.2
LDHA	Lactate dehydrogenase A	NM_001135239.1
LDHB	Lactate dehydrogenase B	NM_001174097.2
MAPK8	Mitogen-activated protein kinase 8	NM_001278547.1
MDH1	Malate dehydrogenase 1	NM_001199111.1
MDH2	Malate dehydrogenase 2	NM_001282403.1
MYC	V-myc avian myelocytomatosis viral oncogene homolog	NM_002467.4
NAMPT	Nicotinamide phosphoribosyltransferase	NM_005746.2
NAPRT1	Nicotinate phosphoribosyltransferase	NM_145201.5
NOX1	NADPH oxidase 1	NM_007052.4
NOX3	NADPH oxidase 3	NM_015718.2
NOX4	NADPH oxidase 4	NM_016931.4
NQO1	NAD(P)H dehydrogenase, quinone 1	NM_000903.2
OGDH	Oxoglutarate (alpha-ketoglutarate) dehydrogenase	NM_001003941.2
PARP1	Poly (ADP-ribose) polymerase 1	NM_001618.3
PC	Pyruvate carboxylase	NM_000920.3
PDHA1	Pyruvate dehydrogenasealpha 1	NM_000284.3
PDK1	Pyruvate dehydrogenase kinase 1	NM_001278549.1
PFKFB1	Phosphofructokinase 2, PFKFB1	NM_001271805.1
PFKM	Phosphofructokinase 1, PFKM	NM_001166686.1
PGAM1	Phosphoglycerate mutase 1	NM_002629.3
PGD	Phosphogluconate dehydrogenase	NM_002631.3
PGK1	Phosphoglycerate kinase 1	NM_000291.3
PGK2	Phosphoglycerate kinase 2	NM_138733.4
PKM	Pyruvate kinase, muscle	NM_001206796.2
PRDX1	Peroxiredoxin 1	NM_001202431.1
PRKAA1	AMP-activated protein kinase, AMPK, catalytic subunit α1	NM_006251.5
PRKAA2	AMP-activated protein kinase, AMPK, catalytic subunit α2	NM_006252.3
RPIA	Ribose 5-phosphate isomerase A	NM_144563.2
SDHA	Succinate dehydrogenase complex, subunit A	NM_001294332.1
SDHB	Succinate dehydrogenase complex subunit B	NM_003000.2
SDHC	Succinate dehydrogenase complex, subunit C	NM_003001.3
SDHD	Succinate dehydrogenase complex, subunit D	NM_003002.3
SLC16A1	Monocarboxylate transporter 1	NM_001166496.1
SLC16A3	Monocarboxylate transporter 4	NM_001206952.1
SLC16A7	Monocarboxylate transporter 2	NM_001270622.1
SLC1A2	Glutamate transporter, high glial high affinity 2	NM_001195728.2
SLC25A5	Adenine nucleotide translocase 2	NM_001152.4
SLC2A1	Glucose transporter 1	NM_006516.2
SLC2A3	Glucose transporter 3	NM_006931.2
SLC5A1	Sodium glucose cotransporter	NM_001256314.1
SLC7A1	Cationic amino acid transporter, y + system	NM_003045.4
SLC9A1	Na^+^/H^+^ proton antiporter	NM_003047.4
SOD1	Superoxide dismutase 1	NM_000454.4
SOD2	Superoxide dismutase 2, mitochondrial	NM_000636.2
TALDO1	Transaldolase	NM_006755.1
TP53I3	Tumor protein p53 inducible protein 3	NM_004881.4
TXN	Thioredoxin	NM_001244938.1
VHL	Von Hippel Lindau	NM_198156.2

To establish the strength of the technique we used the VHL-defective ccRCC cell line SKRC7^46^ and its VHL-complemented variant SKRC7-VHL^HA^ (expressing functional VHL with a haemagglutinin-tag) as a prototypical isogenic cell line pair with different metabolic characteristics: the lack of VHL in SKRC7 results in constitutive stabilization of HIF-1α and HIF-2α and a pseudohypoxic response^39, 46, 47^. Re-introduction of VHL leads to restored HIF1/2 breakdown and normalized expression levels of hypoxia-induced genes.

**Figure 2:**
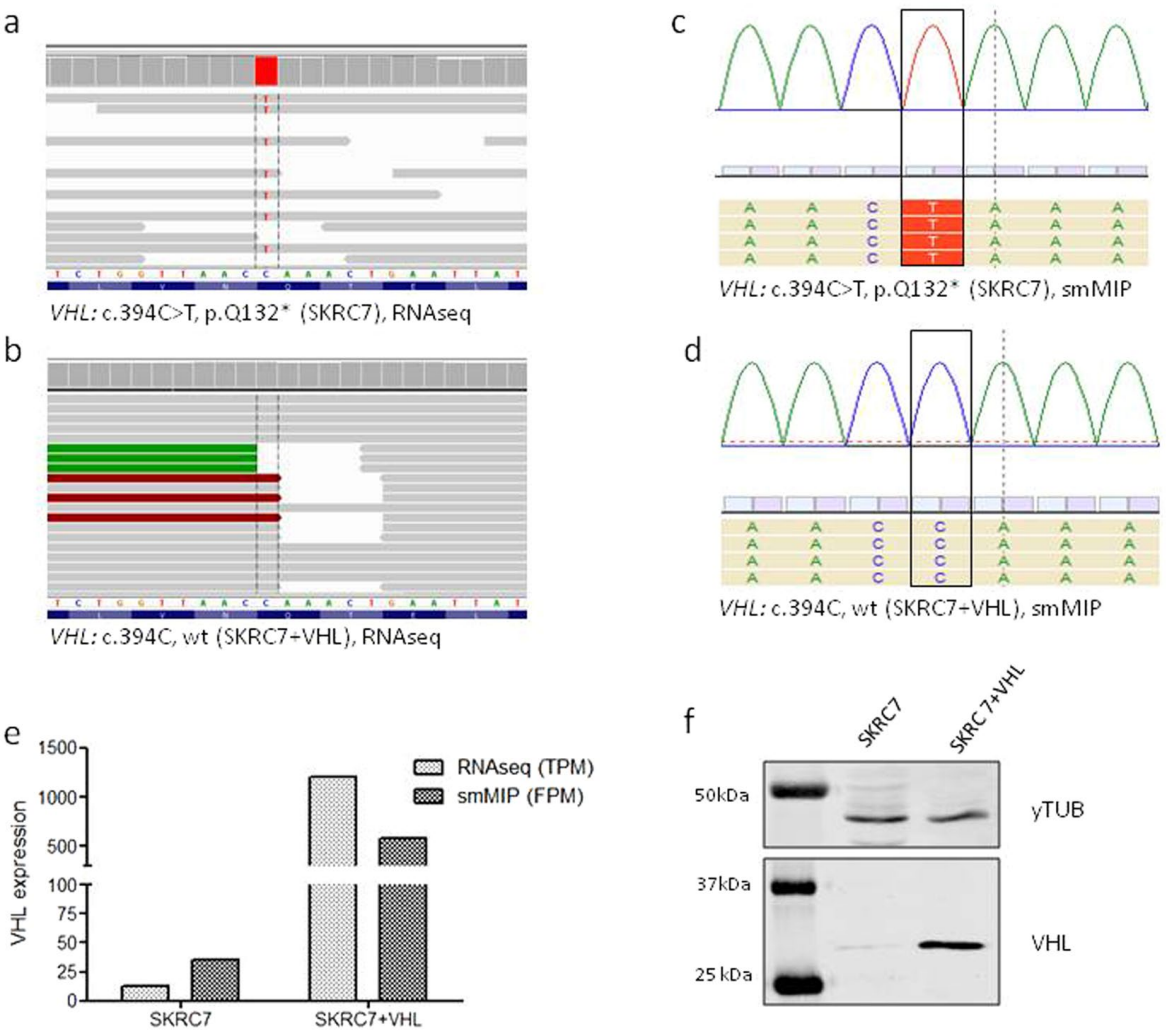
(**a,b**) IGV representation of the VHL locus of SKRC7 and SKRC7-VHL^HA^ cells. BAM files containing whole RNAseq data from these cell lines were loaded into IGV. Note the CAA-UAA mutation, resulting in the VHL ^Q132-stop^ mutation at the protein level. c and d show SeqNext representations of the same VHL locus of SKRC7 (**c**) and SKRC7-VHL^HA^ cells (**d**). (**e**) bar graph showing VHL-related TPM and FPM values of SKRC7 and SKRC7-VHL^HA^. (**f**) Western blot of SKRC7 cells and the VHL-expressing derivative, stained with an anti-HA antibody and an antibody against GAPDH as control house keeping protein. Panel f represents 2 cropped images from different western blots, loaded with the same protein samples, derived from SKRC7 and SKRC7-VHL cells as indicated. The corresponding full blots are presented in Supplementary Figure 2.

Whole RNAseq-derived gene expression data of SKRC7 cells (used here as gold standard) confirmed the presence of a nonsense and functionally inactivating Q132-stop mutation in 100% of VHL transcripts (Fig. 2a) whereas only wtVHL transcripts were detected in the SKRC7VHL^HA^ (Fig. 2b), and this was readily reproduced in the smMIP assay (Fig. 2c,d). Overexpression of VHL transcripts in SKRC7-VHL^HA^ cells was observed both in whole RNAseq datasets (expressed as Transcript per Million, TPM) and in smMIP based datasets (expressed as Fragment per Million, FPM) (Fig. 2e). Overexpression was also seen on the VHL protein level (see western blot in Fig. 2f).

### Optimization of library preparation

Using an initial set of 642 smMIPs, covering the 104 transcripts of interest for this study (see Table 1), we tested our protocol of library preparation with 50 ng of hexamer-primed cDNA on 13 different RNA samples (cell line -and xenograft derived) of which also whole RNAseq datasets were available. A 25-cycle PCR with barcoded primers on the circularized smMIP library yielded PCR fragments of the expected size of 266 bp (not shown). Illumina NextSeq sequencing of the libraries generated with RNA from SKRC7 and SKRC7-VHL cells, yielded 286,000 and 69,000 annotated unique reads respectively (corrected for PCR-amplicates based on the unique molecule identifier [UMI] sequence in the smMIP), which is in the range of other samples run with the same smMIP panel (not shown). For most transcripts, performance of individual smMIPs varied greatly, a known phenomenon also in DNA smMIP NGS^45^ (see example in Table 2, showing FPM values for 10 different smMIPs designed against the VHL transcript in both cell lines). This was a priori reason to include at least 5 smMIPs per gene transcript in our panel, allowing transcriptome analysis using mean FPM values for each transcript. This number was a trade-off between generating expensive, large panels which would yield in part futile and irrelevant data, and too small panels resulting in under- or overestimation of transcript levels.

**Table 2:** variability in FPM values for different smMIPs designed to detect VHL.

smMIP	SKRC7	SKRC7 + VHL
VHL_1	6.98	0.00
VHL_2	20.94	6293.31
VHL_3	0.00	115.74
VHL_4	317.58	54614.37
VHL_5	0.00	1504.61
VHL_6	55.84	14120.17
VHL_7	125.64	16680.89
VHL_8	27.92	0.00
VHL_9	6.98	1157.39
VHL_10	62.82	14.47

**Figure 3:**
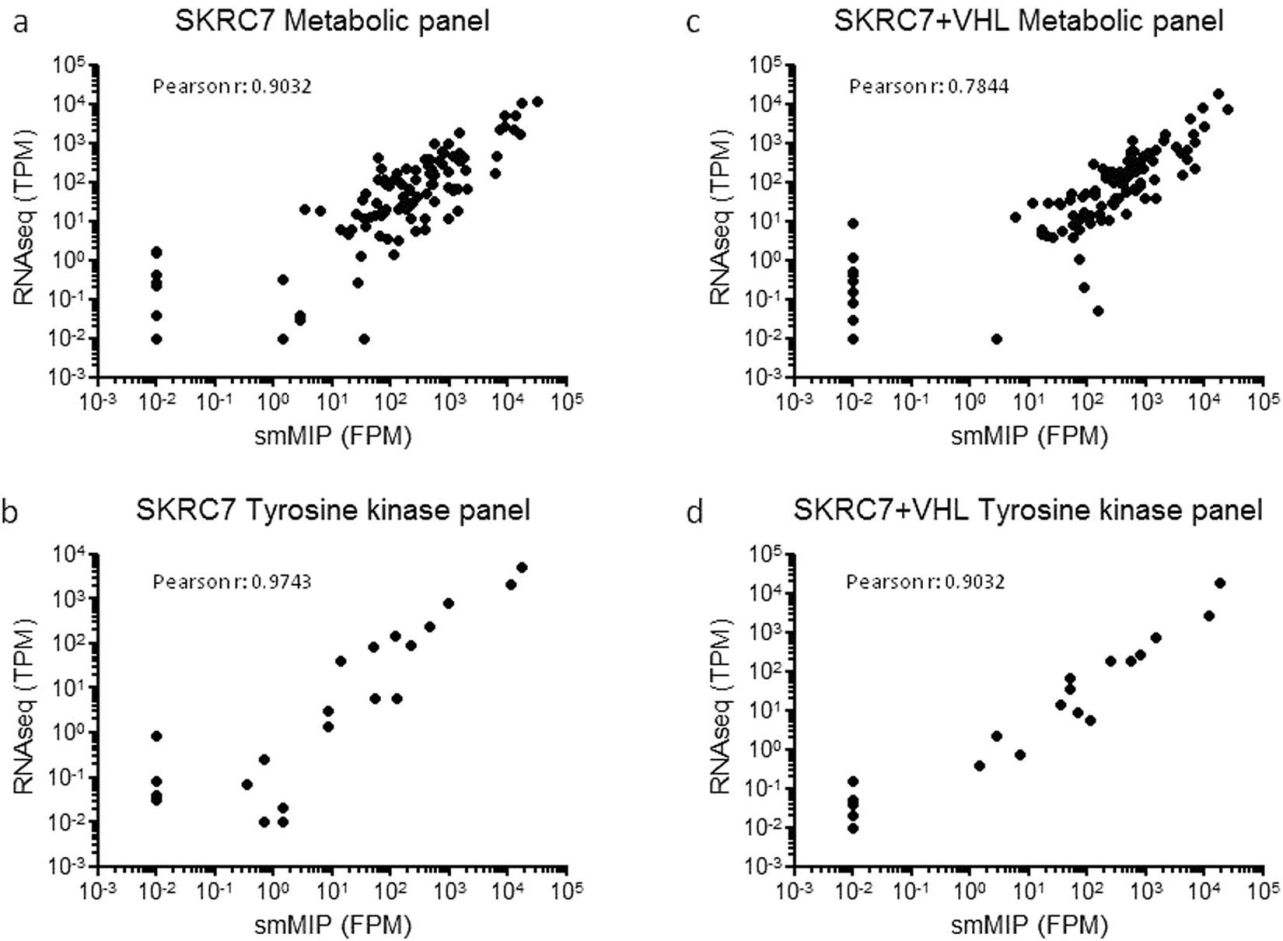
smMIP-based targeted RNA sequencing correlates well with whole transcriptome RNAseq. Mean smMIP-based metabolic FPM levels (**a,c**) and tyrosine kinase transcript FPM levels (**b,d**) were plotted to TPM levels of the same transcripts, extracted from whole RNAseq data. Note that the transcripts with very low FPM values (10^−2^FPM) were not detected in the RNAseq dataset. We included these transcripts in these analyses although they may have lowered the Pearson coefficient.

First we compared the targeted smMIP RNAseq dataset to a whole transcriptome RNAseq dataset (considered as gold standard), performed on different RNA isolates from the same cell lines. The whole RNAseq dataset consisted of 3.2 × 10^7^ and 3.4 × 10^7^ reads, assigned to 44,503 different transcripts for SKRC7 and SKRC7-VHL^HA^, respectively. For each transcript of interest, TPM values from the whole RNAseq dataset were plotted against mean FPM values from smMIP analyses. When performed separately for metabolic transcripts and tyrosine kinase transcripts, these analyses gave correlation coefficients of 0.903 and 0.974, respectively, for SKRC7 (Fig. 3a,b) and 0.784 and 0.903, respectively, for SKRC7-VHL^HA^ (Fig. 3c,d), suggesting that, as expected, expression of metabolic genes is subject to more biological variation than of tyrosine kinases. Plotting whole transcriptome RNAseq data against unique reads obtained with the best performing smMIP per transcript, or the median of unique reads for each transcript (to prevent bias by non- or poor-performing smMIPs) did not improve this correlation (not shown).

One of the appealing characteristics of targeted RNAseq using smMIPs is that panels can be expanded to detect additional transcripts. To test how this affects the outcome of the assay, we added to our initial panel 222 smMIPs for targeted detection and sequencing of other transcripts of interest and re-performed the assay using newly isolated RNA from the same cell lines. Relative levels of transcripts within samples correlated well between assays with the initial and the expanded smMIP set (SKRC7: r = 0.903, SKRC7-VHL^HA^: r = 0.876).

### Functional validation of targeted smMIP data

**Figure 4:**
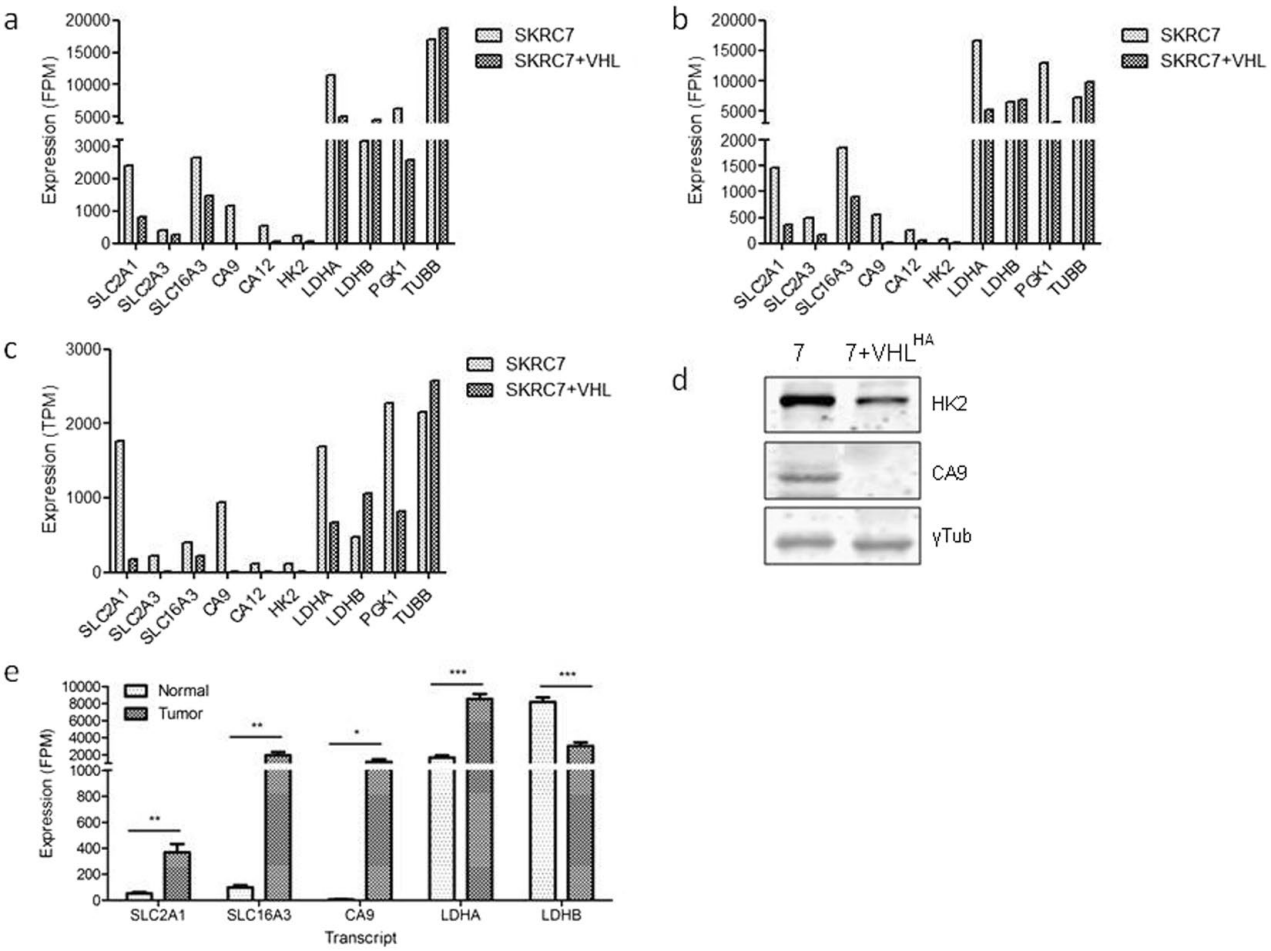
SmMIP-based targeted RNAseq reveals decreased expression levels in SKRC7-VHL cells of glycolysis related genes a.o. SLC2A1, CA9, HK2 and LDHA in two independent duplicate experiments (**a,b**). Relative values were comparable to those obtained from whole transcriptome RNAseq analysis (**c**), which is in agreement with the correlation shown in Fig. 3. Differences in expression levels were validated on the protein level for HK2 and CA9, using tubulin as house keeping control (Fig. 4d). Gene transcript levels of hypoxia inducible genes are very high in surgically obtained clear cell renal cell cancer samples, relative to peritumorally obtained normal kidney tissue (**e**, *p = 0.01; **p < 0.003, ***p < 0.0003, Students’ T-test) (Fig. 4e).

Having confirmed the validity of the smMIP dataset, we analyzed expression levels of genes involved in metabolism in SKRC7 and SKRC7-VHL^HA^ cells. Figure 4a and b show two biological duplicates of smMIP-based mean FPM values for a number of transcripts involved in glycolysis. Expression of HIF target genes glucose transporter 1 and 3 (SLC2A1 and SCL2A3), monocarboxylate transporter MCT4 (SLC16A3), carbonic anhydrases 9 and 12 (CA9, CA12), hexokinase 2 (HK2), lactate dehydrogenase A (LDH-A) and phosphoglycerate kinase (PGK1) were significantly and reproducibly reduced in SKRC7-VHL^HA^ cells relative to SKRC7 cells (Fig. 4a,b), in line with data obtained from whole transcriptome RNA seq data (Fig. 4c). Relative expression levels of CA9 and HK2 transcript levels were further confirmed on the protein level (Fig. 4d). The strong reduction of CA9, HK2 and LDHA, all target genes of HIF, was in line with expectations.

We next tested the assay on clinical renal cell cancers. Of four tumor nephrectomies we sampled normal kidney tissue and matched tumor for smMIP profiling. Striking and highly significant differences were found between normal kidney and tumor tissue, with high levels in tumor samples of transcripts of HIF target genes *SLC2A1* (encoding Glucose transporter GLUT-1), monocarboxylate transporter *SLC16A3* (MCT4), *CA9, and LDHA*, and low levels of *LDHB* in contrast to matched normal kidney (Fig. 4e). Furthermore, the assay readily detected a somatic stop mutation in VHL in renal cell cancer, but not in the corresponding normal kidney tissue (Table 3).

**Table 3:** Somatic mutation (frame shift resulting in a stop) in VHL in renal cell cancer, but not in peritimoral non-neoplastic tissue.

	Gene	Name	Nuc Change	Coverage	AA Change	Hint	c. HGVS	p. HGVS	Weighting
Kidney	VHL								
Renal cancer	VHL	VHL	CG (het)	9% (22) [9% (11) / 9% (11)]	[STOP] AA 130 (E2/48)	RF changed	c.246_247delCG	p.Val83Argfs*48	distinct

### Variant detection

**Figure 5:**
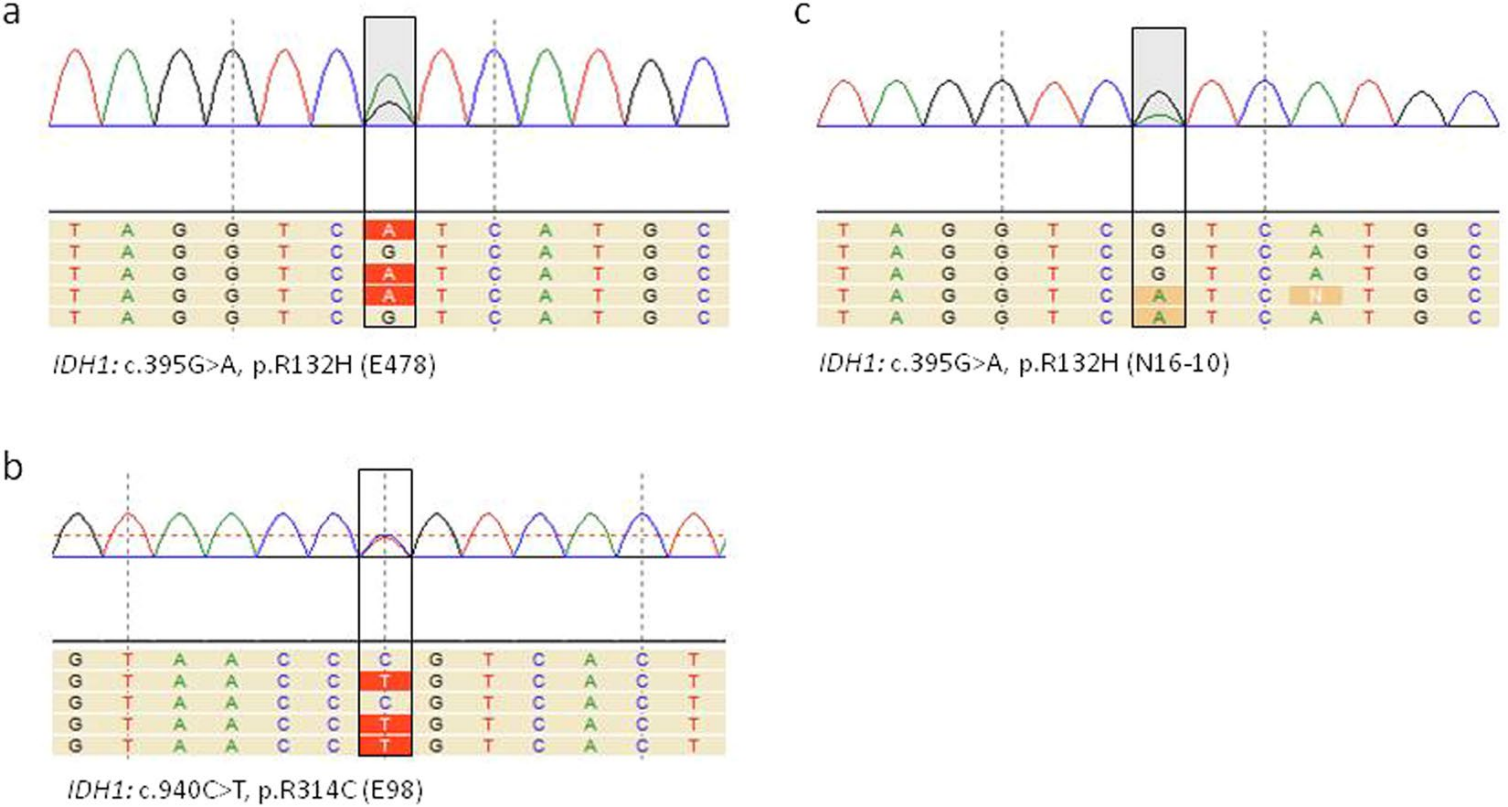
smMIP-based targeted RNA next generation sequencing can be used for adequate variant calling. Shown are the loci containing the IDH1-R132H mutation in E478 xenografts (**a**) and in a clinical grade III astrocytoma (**c**), this mutation was confirmed by genetic analysis), whereas the IDH1-R314C mutation in E98 cells could also be identified (**b**).

To investigate whether smMIP based RNAseq allows efficient detection of single nucleotide variants (SNVs), we performed variant calling of the smMIP library in SeqNext software. Several heterozygous and homozygous variants were detected that could be validated in the whole RNAseq dataset (see VHL example in Fig. 2c and d). We then further validated the sensitivity of the assay to detect SNVs (called a variant in relation to reference genome hg19) and performed smMIP analysis on RNA, isolated from the IDH1^R132H^ mutant oligodendroglioma line E478^48^ and the astrocytoma cell line E98, in which we previously identified a novel mutation in IDH1 (IDH1^R314C^)^35^. Both mutations were identified (Fig. 5a,b). Finally, a patient glioma with a genetically confirmed IDH1^R132H^ mutation was subjected to the assay. Again, the mutation was readily detected (Fig. 5c).

## Discussion

Currently, identifying metabolic pathways in tissues is only feasible in a research setting, inferring metabolic pathways from metabolite concentrations as detected with *in vivo* ^1^H-, ^13^C- or ^31^P-based magnetic resonance spectroscopic imaging^28, 49^ or mass spectrometry^50–52^. These techniques cannot be applied for routine patient diagnostics. Although profiling of the activity of metabolic genes cannot be considered as equivalent to metabolomic analysis or metabolic flux analysis, we here show that smMIP-based targeted RNA sequencing can be applied to any tissue and does yield reliable functional information, as demonstrated here by analysis of effects of VHL-reconstitution in ccRCC. We further present evidence that profiling of clinical renal cell cancer tissues as expected shows high levels of hypoxia-induced genes suggesting extensive glycolysis, while also unambiguously revealing the presence of VHL mutations, all in line with known biology of these cancers. Similarly, the assay unambiguously identifies the IDH1-R132H mutation in glioma, with 50% mutant/wild type reads, confirming heterozygosity for this mutation.

SmMIP-based RNA NGS has advantages over whole RNAseq. It yields workable data at a fraction of the cost of whole RNAseq, and smart positioning of extension and ligation target sequences allows the design of splice-variant-specific smMIPs. Furthermore, smMIP sets can be extended at any time with novel smMIPs of interest without affecting performance. Efficacy of different smMIPs that are designed to detect the same transcript is variable. As for now, it is difficult to discriminate between low intrinsic efficiency of a smMIP and low abundance of the target sequence (e.g. in case of pseudo-exons), and these issues should be resolved for individual genes. Related to this it must be realized that reliable detection of mutations or splice variants depends on the efficacy of smMIPs covering the area of interest. This may present a problem when trying to detect mutations in VHL using smMIPs 1,8 or 10. A way of improvement could be to rebalance the smMIP concentrations in the capture pool for smMIPs having low capture efficiency. With the generation of a database containing targeted RNAseq datasets from large numbers of cancers with the same smMIP panel it will become possible in the future to assign a value of relevance to individual smMIPs.

Targeting of cancer-specific metabolic pathways is gaining importance in cancer research. Since decades glycolysis has been considered the predominant metabolic pathway in cancer, but it is increasingly clear that tumors can also thrive on other sources, such as amino acids, acetate and fatty acids. Identification of the fuel-processing pathways that represent metabolic Achilles heels in cancer is important to apply metabolic inhibitors in a personalized fashion. SmMIP-based transcript profiling may be a highly relevant alternative with added value in the field of cancer diagnostics as it can identify metabolic Achilles heels by simultaneously measuring relative gene expression levels and detecting variants. When combined with smMIP sets that detect actionable mutations in oncogenes or tumor suppressor genes, personalized treatment protocols may be optimized by including inhibitors of the most predominant metabolic pathways (e.g. 3-bromopyruvate, dichloroacetate for glycolysis^53–55^, 6-aminonicotinamide for the pentose phosphate pathway^20^, epigallocathechin-3-gallate for glutaminolysis^56, 57^, metformin for mitochondrial oxidative phosphorylation^58–60^), cerulenin for fatty acid oxidation and lipid synthesis^61^).

## Materials and Methods

### Cell lines

The cell line SKRC7 is derived from a primary human ccRCC and has been described before^46^. Cells were cultured in RPMI 1640 (Lonza Group, Switzerland) supplemented with 10% fetal calf serum (FCS) (Gibco, Thermo Fisher Scientific, Waltham, MA, USA) and 40 µg/ml gentamycin (Centrafarm, Etten-Leur, The Netherlands). An isogenic SKRC7 cell line expressing a functional haemagglutinine (HA)-tagged VHL (SKRC7-VHL^HA^) was created by transfection with pcDNA3.1-VHL^HA^ followed by selection of stable transfectants in the same medium with 400 µg/ml geneticin (Gibco, Thermo Fisher Scientific, Waltham, MA, USA). The patient-derived glioma xenograft models E478 and E98 have been described before^48, 62^.

### Patient material

Use of patient material for this study was approved by the local ethical committee of Radboudumc, and involved prior informed consent. All methods were performed in accordance with the guidelines for use of human tissue of the Radboudumc. Tissue was analyzed in a to the researcher team anonymized manner. Surgically obtained tissue from a patient with a grade III astrocytoma was snap frozen in liquid nitrogen. From four patients suspected for clear cell renal cell cancer, tissue was collected shortly after tumor nephrectomy and snap frozen.

### RNA and cDNA preparation

Total RNA was isolated from sections of snap-frozen E478 xenograft tissue, human tumor tissues and from 80% confluent SKRC7 and SKRC7-VHL^HA^ cells using TRIzol reagent (Life Technologies, ThermoFisher Scientific, Waltham, MA, USA) according to the manufacturers’ instructions. RNA quality was estimated based on relative levels of 28S, 18S and 5S rRNA bands on agarose gel and with Bioanalyzer assays (Agilent Technologies, Amstelveen, The Netherlands). RNA was reverse transcribed to cDNA using Superscript II reverse transcriptase (Invitrogen, ThermoFisher Scientific, Waltham, MA, USA) and random hexamer primers (Promega, Madison, WI, USA) according to standard protocols. Next, cDNA was purified using the NucleoSpin Gel and PCR Clean-up kit (Macherey-Nagel, Düren, Germany). For quality control, cDNA was subjected to PCR for reference gene hydroxymethylbilane Synthase (HBMS) with forward primer HMBSFw (5′-CTGGTAACGGCAATGCGGCT-3′) and reverse primer HMBSRv (5′-TTCTTCTCCAGGGCATGTTC-3′) using AmpliTaq Gold 360 master mix (Applied Biosystems, ThermoFisher Scientific, Waltham, MA USA).

### Whole transcriptome RNAseq analysis

High quality RNA with RIN scores >8 was subjected to whole transcriptome RNAseq at the genomics core facility of the Netherlands Cancer Institute according to standard protocols. Sequencing was performed on an Illumina Hiseq and yielded 30–50 million reads per sample (paired end sequencing protocol). The dataset was analyzed using the ‘Tuxedo’ protocol; reads were mapped against the RefSeq human genome (GRCh37.55) with TopHat and final transcript assembly was done with the Cufflinks package^63^. FeatureCount was used on the BAM files to extract gene counts which were then transformed to transcript per million mapped reads (TPM) to obtain relative expression values. Occurrence of single nucleotide variants was visualized in the Integrated Genomics Viewer browser (IGV, the Broadinstitute).

### smMIP design

The technique of targeted RNAseq using smMIPs is depicted in Fig. 1. It is based on the hybridization of an extension and ligation probe, joined by a backbone sequence, in an inverted manner to a cDNA of interest, followed by gap-filling/ligation and PCR. SmMIPs against the antisense strand of 104 predicted transcripts (UCSC human genome assembly hg19) were designed based on the MIPgen algorithm as described by Boyle *et al*.^64^. Whenever possible, smMIPs were designed with ligation and extension probes located on adjacent exons to prevent contribution of smMIP probes that hybridize to potential contaminations of genomic DNA. Transcripts of interest were encoding enzymes and transporters functioning in various metabolic pathways, including lipid metabolism, glycolysis, oxidative phosphorylation (OXPHOS), tricarboxylic acid (TCA) cycle, pentose phosphate pathway (PPP), glutaminolysis and control of reductive potential (see Table 1). The smMIP set also contained probes for detection of β-actin and β-tubulin as housekeeping genes, and a number of tyrosine kinases with relevance for cancer. SmMIPs were designed with extension probes of minimum length of 16 nt and ligation probes of minimum length of 18 nt, joined by a constant backbone sequence (40 nt) with a stretch of 8 random nucleotides (unique molecule identifier, UMI) incorporated adjacent to the ligation probe. The UMI is incorporated to reduce all amplicons originating from one individual smMIP to one unique MIP (see below). The length of gap-fill was set at 112 nt. Whereas the design was based on full coverage, for the majority of transcripts 5–10 smMIPS per transcript were included in the panel with the target regions distributed evenly over the reading frame. For 18 transcripts (CS, D-2HGDH, L-2HGDH, FH, IDH1-3A-G, MDH1-2, MYC, OGDH, SDHA-D, VHL) smMIP sets were chosen that covered the full coding sequences.

### Capture and library preparation

Generation of libraries was performed with a procedure adapted from O’Roak *et al*.^65^. In short, 642 smMIPs (IDT, Leuven, Belgium) were pooled at 100 µM/smMIP. The smMIP pool was phosphorylated using T4 Polynucleotide Kinase (New England Biolabs, NEB, Ipswich, MA, USA) in T4 DNA ligase buffer (NEB) at 37 °C for 45 min, followed by inactivation for 20 min at 65 °C. The capture reaction was performed with 50 ng of cDNA and an estimated 8000-fold molar excess of the phosphorylated smMIP pool^45^ in a 25 μL reaction mixture containing Ampligase buffer (Epicentre, Madison, WI, USA), dNTPs, Hemo KlenTaq enzyme (New England Biolabs, NEB, Ipswich, MA, USA) and thermostable DNA ligase (Ampligase, Epicentre). The capture mix was incubated for 10 min at 95 °C (denaturation), followed by incubation for 18 h at 60 °C, during which hybridization and concomitant primer extension and ligation occurs. Directly after this step non-circularized smMIPs, RNA and cDNA were removed by treatment with 10 U Exonuclease I and 50 U of Exonuclease III (both NEB) for 45 min at 37 °C, followed by heat inactivation (95 °C, 2 min). The circularized smMIP library was subjected to standard PCR with 2x iProof High-Fidelity DNA Polymerase master Mix (Bio-Rad, Hercules, CA) with a primer set containing a unique barcoded reverse primer for each sample. Generation of PCR products of correct size (266 bp) was validated on agarose gel electrophoresis, and PCR-libraries from different samples were pooled based on relative band intensity. The pool was then purified using AMPureXP beads (Beckman Coulter Genomics, High Wycombe, UK) according to manufacturers’ instructions. The purified library was run on a TapeStation 2200 (Agilent Technologies, Santa Clara, CA, USA) and quantified via Qubit (Life Technologies, ThermoFisher Scientific, Waltham, MA USA) to assess quality of the library.

Reproducibility of the technique was tested by preparing biological replica libraries, using different RNA preparations from the same cell lines.

### Sequencing and annotation

Libraries were sequenced on the Illumina NextSeq platform (Illumina, San Diego, CA) at the Radboudumc sequencing facility to produce 2 × 151 bp paired-end reads. Reads were mapped to the reference transcriptome (hg19) using the SeqNext module of JSI SequencePilot version 4.2.2 build 502 (JSI Medical Systems, Ettenheim, Germany). The random 8 nt sequence flanking the ligation probe was used to reduce PCR amplicates to one smMIP (unique reads).

### Single nucleotide variant (SNV) calling and expression analysis

All single nucleotide variants (SNVs) called with a minimal variant percentage of 5% detected in at least 5 unique reads (forward and reverse) were selected for further analysis. Variants were annotated and classified into synonymous or non-synonymous. Next, they were validated in whole transcriptome RNAseq data, generated from different RNA isolations from the same cell lines.

Individual read counts for each smMIP were divided by the total read count within a sample and multiplied by 10^6^ resulting in a fragment per million (FPM) value for each smMIP in a sample. We choose for this normalization procedure instead of normalization against housekeeping genes because perfect housekeeping genes do not exist.

### Western blotting

Cell extracts were prepared from SKRC7 and SKRC7-VHL^HA^ cells by solubilizing in RIPA buffer (Cell Signaling) and protein concentrations were determined using BCA assays. 20 µg of protein was separated on 12% SDS-PAGE gels and electroblotted on nitrocellulose. After blocking in Odyssey blocking buffer (1:1 in PBS) membranes were incubated overnight in Odyssey blocking buffer containing antibodies against HK-2 (2867S, Cell signaling technology), CA9 (M75, Dr. Oosterwijk) or γ-tubulin (C20, Santa Cruz Biotechnology, Dallas, TX) as loading control. Antibodies were detected with secondary antibodies conjugated with Alexa680 or DyLight800, and signal was visualized with the Odyssey scanner (LI-COR).

### Statistics

FPM values for each transcript (mean FPM values from all smMIPs targeting one transcript) were correlated with TPM values (transcripts per million values for the same transcript obtained from whole RNAseq data from the same cell lines. For three samples replicate assays were performed. Correlation analyses were performed using GraphPad Prism v.5.03 (GraphPad, San Diego, CA, USA).

### Data availability

Data relating to this manuscript (excel files with FPM values) will be made available to researchers upon request.

## Electronic supplementary material

Supplementary information
