# Supplementary material for: Profiling of the metabolic transcriptome via single molecule molecular inversion probes
Source: Sci Rep. 2017 Sep 12;7:11402. doi: 10.1038/s41598-017-11035-0 (PMC5595890; doi:10.1038/s41598-017-11035-0)
Supplement: Supplementary file 1 — Supplementary information [file 41598_2017_11035_MOESM1_ESM.pdf]

Supplementary file

Profiling of the metabolic transcriptome via single molecule molecular inversion probes

Tessa de Bitter[1], Carlijn van de Water[1], Corina van den Heuvel[1], Carolien Zeelen[1] Astrid Eijkelenboom[1], Bastiaan Tops[1], Egbert Oosterwijk[2], Dimitar Kolev[2], Peter Mulders[2], Mark ter Laan[3], Sanne van Lith[1], William Leenders[1*]

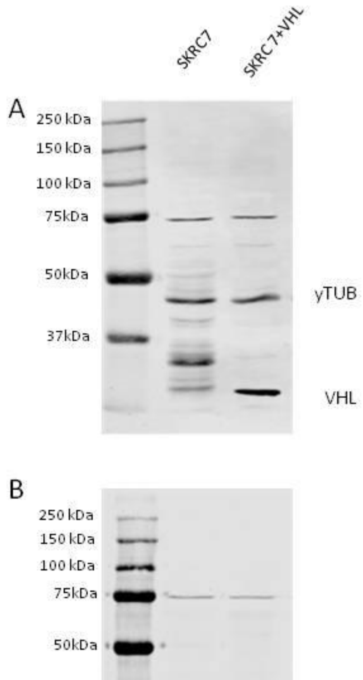

A: western blot containing samples from SKRC7 and SKRC7-VHL-HA cell lines, probed with an antibody against g-tubulin (probed with a 680 nm fluorescent secondary antibody) and an antibody against HA (probed with an 800 nm fluorescent secondary antibody), to detect tagged VHL. The blot was stained in dual color for imaging in the Odyssee (Licor). To prevent confusion about the background signals between 30 and 37 kDa, we repeated the blot with the same samples, but stained for VHL only (panel B).
